# Characteristics of acute febrile illness and determinants of illness recovery among adults presenting to Singapore primary care clinics

**DOI:** 10.1186/s12879-016-1958-4

**Published:** 2016-10-28

**Authors:** Zaw Myo Tun, Mahesh Moorthy, Martin Linster, Yvonne CF Su, Richard James Coker, Eng Eong Ooi, Jenny Guek-Hong Low, Gavin J. D. Smith, Clarence C. Tam

**Affiliations:** 1 Saw Swee Hock School of Public Health, National University of Singapore, Tahir Foundation Building, 12 Science Drive 2, #10-01, Singapore, 117549 Singapore; 2 Duke-National University of Singapore Medical School, 8 College Road, Singapore, 169857 Singapore; 3 London School of Hygiene and Tropical Medicine, Keppel St, London, WC1E 7HT UK; 4 Department of Infectious Disease, Singapore General Hospital, Outram Road, Singapore, 169608 Singapore

**Keywords:** Acute febrile illness, Primary care, Undifferentiated fever

## Abstract

**Background:**

Undifferentiated acute febrile illness (AFI) is a common presentation among adults in primary care settings in Singapore but large gaps exist in the understanding of the characteristics of these patients. We studied clinical and epidemiological characteristics of AFI patients and factors associated with delayed recovery from AFI.

**Methods:**

We performed a secondary data analysis using data from the Early DENgue infection and outcome (EDEN) study on 2046 adult patients presenting at 5 Singapore polyclinics between December 2007 and February 2013 with a history of fever (≥38 °C) for less than 72 h. We used an accelerated failure time model to investigate factors associated with delayed recovery from AFI.

**Results:**

The mean age of patients was 36.6 years, 65 % were male, 51 % were of Chinese ethnicity, and 75 % lived in public housing. Median illness duration was 5 days (interquartile range, 3–7). In multivariable analysis, the unemployed and white collar workers had longer illness duration compared with blue collar workers (time ratio (TR), 1.10; 95 % confidence interval (CI), 1.03–1.17 and TR, 1.08; 95 % CI, 1.02–1.15, respectively). Patients with more symptoms at initial consultation had slower recovery (TR, 1.03 per additional symptom; 95 % CI, 1.02–1.03). Other clinical factors were also associated with longer duration of illness, including use of analgesics (TR, 1.21; 95 % CI, 1.15–1.28); use of cough medicines (TR, 1.14; 95 % CI, 1.08–1.20); use of antibiotics (TR, 1.14; 95 % CI, 1.07–1.21); and hospitalization (TR, 1.59; 95 % CI, 1.39–1.82). Compared to patients with normal WBC count at first consultation, those with low WBC count had slower recovery (TR, 1.14; 95 % CI, 1.07–1.21), while the reverse was observed among patients with high WBC count (TR, 0.94; 95 % CI, 0.88–1.00).

**Conclusions:**

Differences in illness duration among different types of employment may reflect differences in their underlying general health status. Early identification of factors delaying recovery could help triage management in a primary care setting. In-depth characterization of fever etiology in Singapore will improve surveillance and control activities.

## Background

Undifferentiated acute febrile illness (AFI) is a common presentation in primary care settings and has wide-ranging etiologies. It is typically self-limiting with short duration and is presumed to originate from an infectious source [1]. Diagnosis of AFI is usually based on clinical features and management is empirical, due to the broad spectrum of differential diagnoses and lack of suitable point-of-care tests [2, 3].

Most studies on AFI in the Asian continent focus on distinct population subgroups, such as children [4, 5], hospitalized patients [6–10], pregnant women [11], and cases of fever of unknown origin (defined as core body temperature higher than 38.3 °C on several occasions for at least 3 weeks) [12, 13]; studies on AFI in the general adult population are lacking.

Singapore is a Southeast Asian island state situated at the equator with a population of 5.47 million and a high degree of cultural diversity, trade, travel, and population migration. It is a high-income country with a high burden of febrile illnesses, including influenza and dengue [14]. Upper respiratory tract infections, often inducing fever, account for 29 % of all primary care consultations in Singapore in 2010 [15]. However, large gaps exist in the understanding of the clinical and epidemiological characteristics of AFI among adults in Singapore and the Asian region. Identification of risk factors for adverse outcomes in primary care patients would allow triage management of individual cases and timely provision of healthcare at a population level. In this report, we use clinical and epidemiological data collected from patients enrolled in the Early DENgue infection and outcome (EDEN) study between 2007 and 2013 to identify factors associated with delayed illness recovery in febrile adults presenting to primary care services [16].

## Methods

### Study population

Singapore polyclinics are government-subsidized, comprehensive primary care clinics staffed by physicians, nurse practitioners and nurses, equipped with X-ray and laboratory services, and onsite pharmacies. The National Health Survey reported that among adult Singapore residents who consulted primary care providers, 1 in 4 accessed polyclinic services in 2010 [17].

We performed a secondary data analysis using data collected in the EDEN study, which enrolled patients above 17 years of age who presented with a history of fever (≥38 °C) for less than 72 hours at any of 5 Singapore polyclinics [16]. Enrolment took place between December 2007 and February 2013. Demographic and clinical information was collected using standardized forms. Recruited subjects were followed up on 2 separate occasions, at 2–3 days and 4 weeks from the initial visit. Venous blood and/or nasopharyngeal swabs were obtained at the first and second visits for a range of laboratory diagnostics. At the last visit, participants reported the total duration of illness, defined as the self-reported number of days between symptom onset and complete recovery. For the purposes of this analysis, AFI was defined as undifferentiated fever (body temperature ≥38 °C) lasting no longer than 72 hours at first presentation. Hospitalization within 5 days of study enrolment was considered to be a result of the febrile illness. Influenza-like illness (ILI) was classified according to the World Health Organization (WHO) definition [18].

### Statistical analysis

#### Descriptive analysis

Discrete and continuous variables were summarized using the mean (standard deviation, SD) or median (interquartile range, IQR) as appropriate; categorical variables were summarized using frequencies and percentages. We used Kruskal-Wallis rank test to compare the medians of illness duration among categories of explanatory variables. Kaplan-Meier plots based on gender and type of employment were reported.

#### Factors associated with delayed recovery

Cox proportional hazards regression is commonly used to investigate factors associated with time to recovery from AFI, assuming the outcome rates are proportional over time between exposure groups. In our dataset, this proportional hazards assumption was violated for a number of explanatory variables. Instead, we used an accelerated failure time (AFT) methods to model time to recovery directly. In this framework, we investigated models assuming different distribution functions of time to recovery (Weibull, log logistic, log normal and generalized gamma). The model with the distribution that provided the smallest Akaike information criterion (AIC) estimate was determined and reported.

Demographic, clinical and laboratory variables were used as explanatory variables. Variables with a *p*-value of <0.2 from univariable analysis were considered in multivariable analysis. The contribution of individual variables to the multivariable model was assessed using the likelihood ratio (LR) test; variables with a LR test *p*-value <0.05 were retained in the model using forward stepwise exclusion. In multivariable analysis, age group, gender, and polyclinic were included as *a priori* potential confounding variables. The exposure variables considered are described in Table 1. For each factor we estimated the corresponding time ratios (TR) and 95 % confidence interval (CI). A TR smaller than 1 means the factor is associated with longer illness duration, while a TR greater than 1 means the factor is associated with shorter illness duration compared to reference factor. Statistical analysis was performed using Stata 12 (Stata Corp).

**Table 1:** Characteristics of adults with acute febrile illness presenting at the Singapore polyclinics between December 2007 and February 2013

Characteristics (*N* = 2046)	*n* (%)	Median illness duration in days (IQR)	*P value* ^*^
Age groups (years)			
17–24	540 (26.4)	5 (3–7)	0.0002
25–34	567 (27.7)	5 (3–7)	
35–44	333 (16.3)	5 (4–7)	
45–54	295 (14.4)	5 (3–7)	
55–64	210 (10.3)	5.5 (4–7)	
65 and above	101 (4.9)	6 (4–8)	
Gender			
Female	725 (35.4)	5 (4–7)	0.0001
Male	1321 (64.6)	5 (3–7)	
Ethnicity			
Chinese	1083 (51.0)	5 (4–7)	0.0011
Indian	360 (16.9)	5 (3–7)	
Malay	377 (17.7)	5 (3–7)	
Other	303 (14.3)	5 (4–7)	
Missing value	1 (0.0)		
Body mass index			
< 18.5	178 (8.7)	5 (3–7)	0.6460
18.5–22.9	885 (43.3)	5 (3–7)	
23 – 27.4	682 (33.3)	5 (3–7)	
≥ 27.5	301 (14.7)	5 (4–7)	
Migration status			
Singaporean	1286 (62.9)	5 (4–7)	0.0002
Immigrant	100 (4.9)	5 (3–7)	
Missing value	3 (0.1)		
Type of employment			
Blue-collar	1086 (53.1)	5 (3–7)	0.0001
White-collar	457 (22.3)	5 (3–7)	
Other	54 (2.6)	6.5 (4–10)	
Unemployed	447 (21.8)	5 (4–7)	
Missing value	2 (0.1)		
Housing type			
Condominium	81 (3.8)	5 (4–7)	0.0001
Dormitory/Hostel	231 (10.9)	4 (3–6)	
HDB Flat	1588 (74.8)	5 (3–7)	
Landed Property	135 (6.4)	5 (4–7)	
Work site	87 (4.1)	5 (4–7)	
Missing value	2 (0.1)		
Hospitalization as a result of AFI			
No	1986 (97.1)	5 (3–7)	0.0001
Yes	60 (2.9)	10 (7–13)	
Diabetes			
No	1952 (95.4)	5 (3–7)	0.0301
Yes	94 (4.6)	6 (4–8)	
Temperature at initial consultation (°C)			
< 38	660 (32.3)	4 (3–7)	0.0002
38–38.9	999 (48.9)	5 (4–7)	
≥ 39	384 (18.8)	5 (4–7)	
Missing value	3 (0.2)		
WBC count at initial consultation (10^3^ cells/μL)			
< 4 (Low)	213 (10.4)	7 (4–10)	
4–11 (Normal)	1475 (72.1)	5 (3–7)	0.0001
> 11 (High)	345 (16.9)	4 (3–7)	
Missing value	13 (0.6)		
Number of symptoms at initial consultation (excluding fever)			
0–2	290 (14.2)	4 (3–6)	0.0001
3–4	515 (25.2)	5 (3–7)	
5–7	753 (36.8)	5 (4–7)	
8 and more	488 (23.9)	6 (4–7)	
Severity of anemia at initial consultation^a^			
No anemia	1821 (89.0)	5 (3–7)	0.2473
Mild	133 (6.5)	5 (4–7)	
Moderate	54 (2.6)	5 (4–7)	
Severe	12 (0.6)	6 (4.5–10)	
Missing value	26 (1.3)		
Influenza-like illness^b^			
No	1371 (67.0)	5 (3–7)	0.0001
Yes	672 (32.8)	5 (4–7)	
Missing value	3 (0.1)		
Analgesic use			
No	1060 (51.8)	4 (3–7)	0.0001
Yes	753 (36.8)	6 (4–7)	
Missing value	233 (11.4)		
Cough medicine use			
No	1213 (59.3)	4 (3–7)	0.0001
Yes	600 (29.3)	6 (4–7)	
Missing value	233 (11.4)		
Antibiotic use			
No	1510 (73.8)	5 (3–7)	0.0001
Yes	303 (14.8)	6 (4–8)	
Missing value	233 (11.4)		

*AFI* Acute febrile illness, *HDB* Housing Development Board, *IQR* Inter-quartile range

^a^ No anaemia (male, ≥ 13; female, ≥ 12); Mild anaemia (male, 11–12.9; female, 11–11.9); Moderate anaemia (8–10.9); Severe anaemia (<8) in Hb (g/dl) [26]

^b^ILI: combination of symptoms including fever, cough, sore throat

^*^ Kruskal-Wallis rank test *p* value

## Results

From December 2007 to February 2013, 2046 adult patients with AFI were enrolled in the EDEN study (Table 1). The mean age of patients was 36.6 years (SD, 14.8), 1321 (65 %) were male, and 1083 (51 %) were Chinese. The percentage of fever patients aged 65 years and above was substantially higher among Chinese (8.1 %) compared to other ethnic groups (2.6 %, 1.4 %, and 0.7 % among Indians, Malays, and other respectively). In terms of employment, more than half of participants (10866, 53 %) were blue-collar workers, 457 (22 %) were white-collar workers, 54 (3 %) were in other employment categories (including domestic helpers, and self-employed), and 447 (21.8 %) were not employed. The percentage of blue-collar workers was lower among Chinese (41 %) compared to non-Chinese patients (70 %, 53 % and 83 % for Indians, Malays and other ethnicities respectively).

Most patients (75 %) lived in public housing (Housing Development Board (HDB) flats). There were 1286 (63 %) Singaporeans, with other nationalities (including Chinese, Indian, Malaysian, Bangladeshi, and other) each accounting for less than 10 % of the study population. Among all patients, one-third had ILI and 60 (3 %) were hospitalized as a result of their febrile illness.

**Fig. 1:**
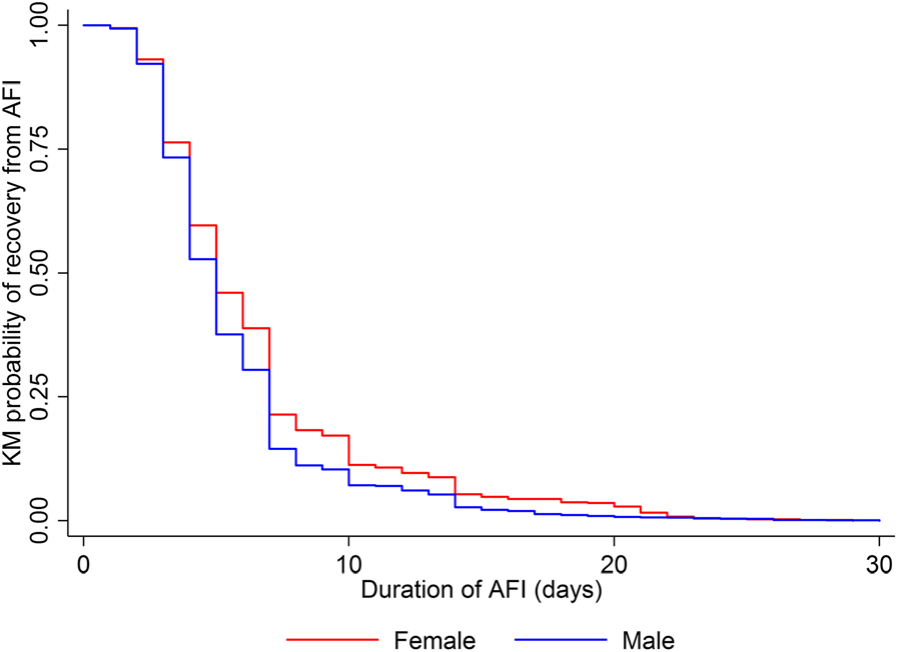
Kaplan-Meier (KM) probability curves of time to acute febrile illness (AFI) recovery based on gender

**Fig. 2:**
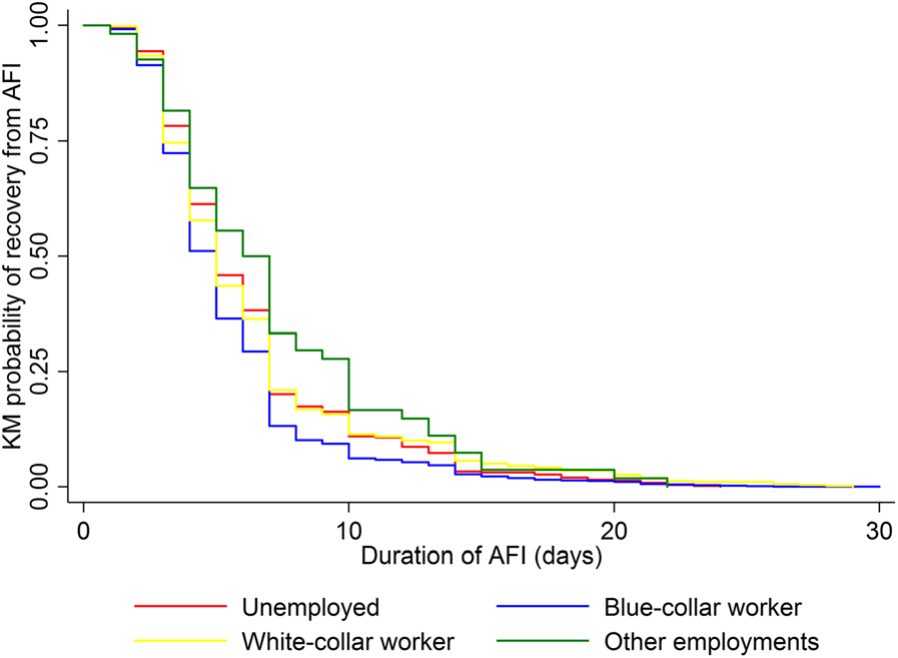
Kaplan-Meier (KM) probability curves of time to acute febrile illness (AFI) recovery based on type of employment

Overall median duration of illness was 5 days (IQR, 3–7), and rate of recovery was 1.18 cases per person-week (95 % CI, 1.13–1.24). A longer illness duration was observed among older age groups, female, Chinese ethnicity (compared to non-Chinese ethnicity) and local Singaporeans (compared to immigrants). On the other hand, a notable shorter illness duration was observed among patients who are blue-collar workers, and those who reside in dormitory or hostel (compared to those living in HDB flats) (Table 1). Figure 1 and 2 show the difference in time to recovery based on gender and type of employment. Duration of AFI reported by patients ranged from 1 to 30 days, and majority of patients recovered in the first 7 days of illness. In Fig. 1, female patients had a slower recovery compared to male patients. In Fig. 2, blue-collar workers had a faster recovery compared to other 3 employment categories.

Among the clinical factors, the illness duration was longer with the increasing number of symptoms at initial consultation. In addition, patients who reported hospitalization, presented with low WBC count at initial consultation, and those who took medications also reported longer duration of AFI (Table 1).

Younger patients were more likely to report a greater number of symptoms at initial presentation; a quarter of fever patients aged 17–24 years reported 8 or more symptoms, compared to 11 % among patients aged 65 years and above (*p* = 0.002). The distribution of symptoms reported by patients at initial consultation is presented in Table 2. Headache, muscle pain, loss of appetite, and taste alteration were the most common symptoms, each reported by more than half of all patients. Vomiting, diarrhoea, swollen lymph nodes, rashes, and bleeding occurred in <10 %. No major differences in symptom distribution were observed by age group.

**Table 2:** Distribution of symptoms at initial consultation among adult patients with acute febrile illness presenting at Singapore polyclinics between December 2007 and February 2013

Symptom	All patients, *n* (%)	*n* (%) of patients with symptom in each age group			*P value*
		17–34 years (*N* = 1107)	35–64 years (*N* = 838)	≥65 years (*N* = 101)	
Headache	1433 (70.0)	815 (73.6)	570 (68.0)	48 (47.5)	<0.001
Muscle Pain	1320 (64.5)	685 (61.9)	583 (69.6)	52 (51.5)	<0.001
Loss of Appetite	1158 (56.6)	644 (58.2)	464 (55.4)	50 (49.5)	0.157
Taste Alteration	1037 (50.7)	548 (49.5)	440 (52.5)	49 (48.5)	0.383
Drowsiness	915 (44.7)	517 (46.7)	365 (43.6)	33 (32.7)	0.017
Joint Pain	810 (39.6)	429 (38.8)	352 (42.0)	29 (28.7)	0.025
Nausea	544 (26.6)	321 (29.0)	210 (25.1)	13 (12.9)	0.001
Red Eyes	470 (23.0)	259 (23.4)	194 (23.2)	17 (16.8)	0.320
Retro Orbital Pain	331 (16.2)	216 (19.5)	111 (13.3)	4 (4.0)	<0.001^a^
Abdominal Pain	323 (15.8)	182 (16.4)	129 (15.4)	12 (11.9)	0.447
Skin Sensitivity	256 (12.5)	140 (12.7)	109 (13.0)	7 (6.9)	0.214
Vomiting	162 (7.9)	92 (8.3)	66 (7.9)	4 (4.0)	0.322^a^
Diarrhea	140 (6.8)	76 (6.9)	58 (6.9)	6 (5.9)	0.933
Swollen Lymph Nodes	101 (4.9)	64 (5.8)	37 (4.4)	0 (0.0)	0.010^a^
Rashes	69 (3.4)	41 (3.7)	25 (3.0)	3 (3.0)	0.692^a^
Bleeding	35 (1.7)	29 (2.6)	6 (0.7)	0 (0.0)	0.003^a^

^a^ Fisher’s exact test was employed on values less than 5

In multivariable analysis, the log-logistic AFT model provided the smallest AIC estimate. Compared to blue-collar workers, longer illness duration was observed among unemployed patients (TR, 1.10; 95 % CI, 1.03–1.17) and white-collar workers (TR, 1.08; 95 % CI, 1.02–1.15). Illness duration increased by 3 % for each additional symptom reported at initial consultation (TR, 1.03 per additional symptom; 95 % CI, 1.02–1.03). Hospitalization was associated with almost 60 % longer time to recovery (TR, 1.59; 95 % CI, 1.39–1.82). Similarly, patients who reported use of analgesics (TR, 1.21; 95 % CI, 1.15–1.28), cough medication (TR, 1.14; 95 % CI, 1.08–1.20), and antibiotics (TR, 1.14; 95 % CI, 1.07–1.21) also had significantly longer illness duration. In addition, compared to patients with normal WBC count at initial visit, those with low WBC count had slower recovery (TR, 1.14; 95 % CI, 1.07–1.21), while the reverse was observed among patients with high WBC count (TR, 0.94; 95 % CI, 0.88–1.00). (Table 3)

**Table 3:** Factors associated with delayed recovery from acute febrile illness among adult patients presenting at Singapore polyclinics between December 2007 and February 2013

Variable^b^	Unadjusted TR^a^ (95 % CI)	*P* value	Adjusted TR^a^ (95 % CI)	*P value*
Type of employment				
Blue-collar workers	1	<0.0001	1	0.0035
White-collar workers	1.10 (1.03–1.16)		1.08 (1.02–1.15)	
Other	1.29 (1.11–1.51)		1.16 (1.00–1.34)	
Unemployed	1.13 (1.07–1.20)		1.10 (1.03–1.17)	
Number of symptoms at initial consultation (excluding fever)	1.04 (1.03–1.05)	<0.0001*	1.03 (1.02–1.03)	<0.0001*
Hospitalization as a result of AFI				
No	1	<0.0001	1	<0.0001
Yes	1.96 (1.72–2.22)		1.59 (1.39–1.82)	
WBC count at baseline (x10^3^ cells/μL)				
< 4 (Low)	1.35 (1.25–1.46)		1.27 (1.18–1.37)	
4–11 (Normal)^$^	1	<0.0001	1	<0.0001
> 11 (High)	0.92 (0.87–0.98)		0.94 (0.88–1.00)	
Analgesic use				
No	1	<0.0001	1	<0.0001
Yes	1.21 (1.15–1.27)		1.21 (1.15–1.28)	
Cough medicine use				
No	1	<0.0001	1	<0.0001
Yes	1.29 (1.22–1.36)		1.14 (1.08–1.20)	
Antibiotic use				
No	1	<0.0001	1	<0.0001
Yes	1.21 (1.13–1.29)		1.14 (1.07–1.21)	

*TR* Time (Illness duration) ratio, *AFI* Acute Febrile Illness, *WBC* White Blood Cells

^a^ A TR smaller than 1 means the factor is associated with longer illness duration, while a TR greater than 1 means the factors is associated with shorter illness duration compared to reference factor

^b^ Age, gender, and polyclinic variables are adjusted for in the model as confounding factors and are not shown

* *p* value for linear trend

## Discussion

This is one of only a few studies to characterize adult patients presenting to primary care with AFI. Our findings indicate that AFI in adults presenting to Singapore polyclinics is relatively mild; all patients recovered from febrile illness within 30 days and a very low percentage of hospitalization was observed. However, the burden of disease is still considerable; 50 % of cases had illness lasting more than 5 days, 36 % used analgesics, 29 % used cough medication, and 15 % used antibiotics.

The availability of data from the EDEN study, comprising a large sample of adult fever patients presenting to public primary care clinics, has provided a unique opportunity to further characterize adult fever patients. In contrast to previous studies that have focused on severely ill hospitalized patients [6–10, 19], our study is based on patients presenting with AFI at community polyclinics in Singapore, where there is a large burden of under-characterized illness. In addition, follow-up of fever patients allowed us to determine characteristics and factors contributing to delayed recovery in this population.

Patients who were blue-collar workers had shorter duration of illness compared to those who were unemployed or white-collar workers. A possible explanation for this is the healthy worker effect; blue collar or manual workers tend to engage in more physically demanding employment and so are likely to have better general health status, allowing them to recover faster. Information on co-morbidities was collected in the EDEN study, but co-morbidities (i.e. malignancy, and ischemic heart disease) were uncommon (<1 %) so we were unable to explore this possibility further.

Compared to older individuals, younger patients presented with more symptoms at initial consultation. A previous EDEN study using an earlier cohort also showed a similar pattern [20]. Non-specific symptoms, such as headache, drowsiness, nausea, retro orbital pain, were reported more frequently by younger patients (Table 2). Some studies have suggested that younger patients have a lower threshold for reporting symptoms [21], although this does not appear to be a consistent finding [22, 23].

Patients who used analgesics, cough medicines, and antibiotics had slower recovery compared to those who did not use these medications. A similar pattern was seen among cases requiring hospitalization. The need for medications and hospitalization is likely to be an indicator of more severe illness requiring longer time for recovery.

Patients with low WBC count at initial consultation had longer duration of illness than those within the normal WBC range. Low WBC count may indicate a sub-optimal immune response to infection. This may reflect a sub-population of patients who mount generally poorer immune responses over a sustained period of time, leading to longer recovery times.

The EDEN study did not include private primary care providers. In Singapore, approximately 80 % of primary care consultations take place at private healthcare providers [17]. In addition, differences in demographic characteristics may exist in the use of public and private care; only 50 % of our polyclinic sample were of Chinese ethnicity, compared to 74 % in the general Singapore population in 2012 [24], and 72 % of fever patients lived in public HDB flats, compared to 82 % in the general population in 2013 [25]. These differences underline the important contribution that private healthcare institutions can make to better understand characteristics of febrile and other diseases, both in Singapore and other settings with parallel public and private health sectors.

The primary focus of the EDEN study was dengue fever. For this reason, fever was the main criterion for enrolment, and certain respiratory symptoms, such as cough, were not systematically recorded, which could have led us to underestimate the frequency of ILI. We sought to partly validate this using additional information captured on use of cough medications.

This analysis focused on undifferentiated fever. This reflects the reality of clinical practice, in which patient management relies primarily on clinical signs and symptoms rather than microbiological diagnosis. For this reason, these findings will be of benefit for the design of policies for the management of fever patients in primary care. Dengue diagnosis in the EDEN study was low (12 %) [20], and further testing of samples using multiplex assays is currently underway, which will enable us to understand the relative importance of different fever pathogens in primary care and further characterize their clinical and epidemiological features.

## Conclusions

Our analyses provide some insights into the clinical characteristics and epidemiology of acute febrile illness in primary care in Singapore. Further studies that include private primary care services are important to give a fuller understanding of acute febrile illness. In addition, improved characterization of the causative agents of fever in Singapore will be useful to improve surveillance and disease control activities.

## Abbreviations

AFI: Acute febrile illness
CI: Confidence intervals
EDEN: Early DENgue infection and outcome study
HDB: Housing development board
ILI: Influenza like illness
IQR: Interquartile range
LR: Likelihood ratio
TR: Time ratio
WBC: White blood cells
